# A Novel Method for Automatic Detection and Elimination of the Jumps Caused by the Instantaneous Disturbance Torque in the Maglev Gyro Signal

**DOI:** 10.3390/s23052763

**Published:** 2023-03-02

**Authors:** Yiwen Wang, Zhiqiang Yang, Ji Ma, Zhen Shi, Di Liu, Ling Shi, Hang Li

**Affiliations:** 1 School of Geology Engineering and Geomatics, Chang’an University, 126 Yanta Road, Xi’an 710054, China; 2 School of Natural Resources and Surveying, Nanning Normal University, 175 Mingxiu East Road, Nanning 530001, China

**Keywords:** maglev gyro total station (GAT), instantaneous disturbance torque, north-seeking gyroscope, heuristic segmentation algorithm, two-sample Kolmogorov-Smirnov test, autocorrelation function

## Abstract

The signal measured by the maglev gyro sensor is sensitive to the influence of the instantaneous disturbance torque caused by the instantaneous strong wind or the ground vibration, which reduced the north-seeking accuracy of the instrument. To address this issue, we proposed a novel method combining the heuristic segmentation algorithm (HSA) and the two-sample Kolmogorov-Smirnov (KS) test (named HSA-KS method) to process the gyro signals and improve the north-seeking accuracy of the gyro. There were two key steps in the HSA-KS method: (i) all the potential change points were automatically and accurately detected by HSA, and (ii) the jumps in the signal caused by the instantaneous disturbance torque were quickly located and eliminated by the two-sample KS test. The effectiveness of our method was verified through a field experiment on a high-precision global positioning system (GPS) baseline at the 5th sub-tunnel of the Qinling water conveyance tunnel of the Hanjiang-to-Weihe River Diversion Project in Shaanxi Province, China. Our results from the autocorrelograms indicated that the jumps in the gyro signals can be automatically and accurately eliminated by the HSA-KS method. After processing, the absolute difference between the gyro and high-precision GPS north azimuths was enhanced by 53.5%, which was superior to the optimized wavelet transform and the optimized Hilbert-Huang transform.

## 1. Introduction

The gyro total station is a north-seeking instrument that integrates a gyroscope and a total station to measure the geographic north azimuth by sensing the rotation of the earth [1,2]. Since it overcomes the defect that global positioning system (GPS) signals are hardly received in some scenarios, it has been widely used in tunnel breakthroughs, mining, subway construction, weapons guidance, missile launch, and other fields [3,4].

Previous studies have suggested that the accuracy of the gyroscope is not only affected by the error of the gyro sensor but also by external environmental factors, such as ground vibration, wind vibration, temperature, etc. [5,6]. However, the conventional suspension-tape gyroscope is susceptible to being disturbed by environmental interference due to the north-seeking scheme of tracking scattered points [2].

In 2008, a new type of high-precision maglev gyro total station (GAT) based on magnetic suspension-supporting and non-contact photoelectric torque feedback technology was jointly designed and developed by the Chang’an University and China Space Age Electronics 16th Research Institute in Xi’an China. GAT monitors the state of the gyroscope in all environments in real time by dynamically measuring 20,000 sets of stator and rotor current values of the torquer at one north-seeking position. By effectively separating the environmental noise from the measured GAT signal through modern data processing methods, the anti-interference capability of the instrument as well as the north-seeking accuracy can be further improved [7,8]. The nominal north-seeking accuracy of the GAT was reported to 3.5″ with a duration of 8 min [2]. Since the GAT was developed, it has been widely used in several major underground projects in China, such as the Qinghai-Tibet railway tunnel, the immersed tunnel of the Hong Kong-Zhuhai-Macao Bridge, and the Qinling water conveyance tunnel of the Hanjiang-to-Weihe River Diversion Project [8].

Previous studies have proved that signal analysis and processing methods were effective for processing GAT signals and further improving the north-seeking accuracy [5,7,9]. Ma et al. [10] divided the GAT signal into four typical types: steady, periodic, jitter, and jumping signals. The characteristics of each type were analyzed in the time and frequency domains using the 3D time-frequency spectra. Previous studies have suggested that the steady GAT signal of the typical types mentioned above is an ideal type of signal [9,10]. For the periodic and jitter GAT signals, corresponding processing methods were proposed by Wang et al. [9] and Liu et al. [11], respectively. However, no specific method is available for processing the jumping GAT signals measured in the environment affected by the instantaneous disturbance torque, such as the instantaneous strong wind, the ground vibration caused by the rapidly passing vehicle and mine blasting, etc. [8].

Methods for processing jumping signals measured by other types of gyroscopes have been studied by many researchers. Peesapati et al. [12] proposed a fiber-optic gyroscope filtering algorithm combining the Kalman filter and adaptive moving average technique, which can effectively denoise the jumping signal. Song et al. [13] proposed a method to efficiently sense the impact components in the microelectromechanical system gyroscope signal using the sliding kurtosis contribution coefficient. However, the signal characteristics of the GAT are significantly different from their gyroscopes. Therefore, the effectiveness of using their method directly for processing the jumping GAT signal will be significantly reduced. The mainstream processing methods for GAT signals are the optimized wavelet transform (WT) and the optimized Hilbert-Huang transform (HHT) proposed by Ma et al. [10] and Zhang [14], respectively. However, these methods are not specific for the processing jumping GAT signals; therefore, their effectiveness needs further validation. Meanwhile, previous scholars have not provided a specific basis or method for determining the beginning and ending epochs of the jumps in the GAT signal, and the discrimination is mainly based on experience [15].

The key problem lies in how to detect the change points in jumping GAT signals accurately and automatically. The traditional change point detection methods for detecting stationary and linear signals, such as the sliding T-test [16], sliding F-test [17], Mann-Kendall test [18], Grammer method [19], etc., have been widely used to detect the change points in various signals [20]. However, the jumping GAT signals show non-linear and non-stationary characteristics due to the influence of the instantaneous disturbance torque [8]. Therefore, it is not valid for these methods to detect change points in GAT signals accurately. The heuristic segmentation algorithm (HSA) proposed by Bernaola-Galván et al. [21,22] was introduced in our study to detect the change points in jumping GAT signals. By dividing the original signal into several subsequences with different mean values, the inefficiency of the traditional method for detecting change points in non-stationary signals is overcome [23]. Therefore, HSA has been widely applied to the change point detection of non-stationary signals in meteorology, hydrology, environmental science, deformation monitoring, and other disciplines [24,25,26].

Previous studies have demonstrated the accuracy of HSA in signal change point detection and identification [19]. However, after detecting all the potential change points in the GAT signal, how to further determine the intervals where the jump occurs is still a problem that needs to be solved. To address the above issues and improve the robustness and environmental adaptability of the GAT, a novel method combining HSA and the two-sample Kolmogorov-Smirnov (KS) test (named HSA-KS method) was proposed in this study. Therefore, the main objectives of this paper were: (i) to propose a method for automatically detecting and eliminating the jumps caused by the instantaneous disturbance torque in the GAT signal; (ii) to validate and prove the advantage of the proposed method by a field experiment.

## 2. Materials and Methods

### 2.1. North-Seeking Principles of the GAT

The signals measured by the GAT are dynamic photoelectric parameters: the stator and rotor current values of the torquer. Based on the magnetic suspension-supporting and non-contact photoelectric torque feedback technology, GAT applies a “reverse torque” in the horizontal direction that is equal to the “north-seeking pointing torque” but opposite in direction. In this case, the torques of the gyro sensor cancel each other out, and the gyro sensor is in a relatively stable state [2]. Here, the real-time reverse torque $M_{i}^{\prime }$ can be measured by collecting the stator and rotor current values of the torquer.

(1) $$M_{i}^{\prime }=k\times I_{R_{i}}\times I_{S_{i}}$$

where *k* is a coefficient whose value depends on the system, $I_{R_{i}}$ is the collected real-time rotor current value of the torquer, and $I_{S_{i}}$ is the collected real-time stator current value of the torquer. Considering the accuracy (3.5″) and duration of the north-seeking, a default measurement of *N* = 20,000 sets of rotor and stator current values at one north-seeking position was set. The mean torque $\overset{¯}{M}$ is calculated as the result of the reverse torque at the current north-seeking position.

(2) $$\overset{¯}{M}=\frac{k\sum_{i=1}^{N}I_{S_{i}}I_{R_{i}}}{N}$$

Subsequently, the final gyro north azimuth $\alpha_{T}$ can be calculated by the mean torque $\overset{¯}{M}$ as follows [8,15].

(3) $$\alpha_{T}=\arcsin\frac{\overset{¯}{M}}{H\omega_{e}\cos\varphi }$$

where H is the moment of inertia of the gyro, $\omega_{e}$ is the rotational angular velocity of the earth, and φ is the local latitude.

The above equations indicate that the key to the north seeking of the GAT is the accurate measurement of the stator and rotor current values of the torquer at a relatively stable state. Previous studies have suggested that the stator current values of the GAT are insensitive to the influence of the environment. The stator current signals exhibit high stability with a normal distribution under different environments [2,7].

However, the rotor current signals are highly sensitive to environmental factors. This is because the rotor of the torquer is positioned beneath the gyro sensor, which is different from the stator of the torquer that is fixed to the inner shell of the instrument. When the GAT is subjected to the disturbance torque caused by the external environment, the stable state of the gyro sensor is disrupted, resulting in a shift in the real-time collected rotor current signal. The signal collection process of the GAT may be affected by the instantaneous disturbance torque, such as the instantaneous strong wind, the ground vibration caused by the rapidly passing vehicles and mine blasting, etc. After the GAT is affected by the instantaneous disturbance torque, the stable state of the gyro sensor is broken. At this point, the gyro north-seeking pointing torque changes, and the real-time reverse torque output from the torquer cannot completely cancel with the north-seeking pointing torque, resulting in the oscillation of the rotor of the torquer. Controlled by the torque feedback module, the torquer generates the corresponding reverse torque to cancel the influence of the remaining north-seeking pointing torque so that the gyro sensor returns to its original stable state. This process is called the “closed-circuit bias correction” of the GAT. This non-stationary process is reflected in the measured rotor current signal, causing the time series to exhibit a jump [8]. Subsequently, the north-seeking results calculated by the mean algorithm (Equations (2) and (3)) will be biased, which results in a decrease in the north-seeking accuracy of the GAT. It can be seen that unlike the GAT stator current signal with high stability, the GAT rotor current signal often shows non-stationary characteristics. Therefore, only the GAT rotor current signal is processed and referred to as the “GAT signal” in this paper.

### 2.2. HSA-KS Method

#### 2.2.1. Main Principle of HSA Applied to the GAT Signal

The function of HSA is to accurately and automatically detect all the potential change points in the GAT signal affected by the influence of the instantaneous disturbance torque.$\;I_{R}\left(t\right)\;={\left(I_{R_{1}},I_{R_{2}},\cdots ,I_{R_{N}}\right)}^{T}$ is the original GAT signal sequence, and its total number of samples is *N* = 20,000. The specific steps of HSA applied to the GAT signal are as follows:

Step 1: Suppose a sliding pointer is moved from left to right along the signal sequence to be segmented. When the pointer is at the *i*-th epoch of the signal, compute the mean values of the subsequences of the signal to the left of the pointer *μ*_1_(*i*) and the right *μ*_2_(*i*), and the standard deviations of the subsequences of the signal to the left of the pointer *s*_1_(*i*) and the right *s*_2_(*i*). Subsequently, the pooled variance *S_D_*(*i*) of the pointer can be calculated by

(4) $$S_{D}\left(i\right)={\left(\frac{\left(N_{1}-1\right)s_{1}^{2}\left(i\right)+\left(N_{2}-1\right)s_{2}^{2}\left(i\right)}{N_{1}+N_{2}-2}\right)}^{1/2}{\left(\frac{1}{N_{1}}+\frac{1}{N_{2}}\right)}^{1/2}$$

where *N*_1_ and *N*_2_ are the numbers of samples in the left and the right subsequence of the pointer, respectively.

Step 2: The statistical significance of the difference between the mean values of the left and the right subsequence of the pointer can be given by the T-test statistic [27].

(5) $$T\left(i\right)=|\frac{\mu_{1}\left(i\right)-\mu_{2}\left(i\right)}{S_{D}}|$$

The statistic *T*(*i*) is used to quantify the difference between the mean values of the left-side and right-side subsequences. A larger *T*(*i*) indicates that the mean values of both subsequences are more likely to be significantly different [21]. Moving the pointer along the signal from left to right, calculate statistic *T*(*i*) at each epoch and obtain the statistic sequence *T*(*t*) corresponding to the signal to be segmented.

Step 3: *T*(*t*) takes the maximum value *T*_max_ when the pointer is at the *k*-th epoch of the signal, the statistical significance *P*(*T*_max_) can be calculated by the Monte Carlo simulations [21].

(6) $$P\left(T_{\max}\right)={\left[1-I_{\left[\nu /\left(\nu +T_{\max}\right)\right]}\left(\delta \nu ,\delta \right)\right]}^{\gamma }$$

where ν=l−2,⋅δ=0.40,⋅γ=4.19lnl−11.54, and *l* is the total number of samples of the signal to be segmented. $I_{X}\left(a,b\right)$ is the incomplete beta function.

(7) $$I_{X}\left(a,b\right)=\frac{1}{\beta \left(a,b\right)}\int_{0}^{X}t^{a-1}{\left(t-1\right)}^{b-1}dt$$

where $\beta \left(a,b\right)=\int_{0}^{1}t^{a-1}{\left(1-t\right)}^{b-1}dt\;=\frac{\Gamma \left(a\right)\Gamma \left(b\right)}{\Gamma \left(a+b\right)},\Gamma \left(\alpha \right)=\int_{0}^{\infty }e^{-t}t^{\alpha -1}dt$.

Step 4: If the difference in mean values between the left and right part of the signal is statistically significant, i.e., if *P*(*T*_max_) is larger or equal to a threshold *P*_0_ (typically set to 0.99), then the signal is segmented into two subsequences at the epoch where the current pointer is located. At this time, a change point is detected at this segmentation epoch. Otherwise, the signal is not segmented. If the signal is segmented, we continue iterating step 1 to step 3 recursively on each subsequence until all *P*(*T*_max_) is smaller than *P*_0_ or the total number of samples of the obtained subsequence *l* is smaller than a minimum size *l*_0_. To detect the entire potential change points in the GAT signal, in this paper, we set *l*_0_ = 0.

Through the processing of the HSA, the original GAT signal *I_R_*(*t*) is segmented into *m* non-overlapping subsequences $I_{R_{1}}\left(t\right),I_{R_{2}}\left(t\right),\cdots ,I_{R_{m}}\left(t\right)$ with different mean values. The segmentation epochs are the detected change points in the GAT signal, and *m* − 1 is the number of the change points.

#### 2.2.2. Determination of the Categorical Stationary Subsequence

After segmenting the original GAT signal into individual subsequences in the previous section, we need to determine which of them are the stationary subsequences and which are the jumping subsequences. We identify a “categorical stationary subsequence” (CSS) *I*_0_(*t*) in the *m* subsequences as the benchmark. The CSS *I*_0_(*t*) needs to meet the following two requirements:

(i) The CSS *I*_0_(*t*) should contain enough samples. Since the stationary subsequence in the GAT signal is relatively longer than the jumping subsequence caused by the instantaneous disturbance torque, in our method, we required that *I*_0_(*t*) should contain more than 4000 samples.

(ii) The standard deviation of CSS *I*_0_(*t*) should be small. This is because the standard deviation of the stationary subsequence in the GAT signal is relatively smaller than the jumping subsequence.

In summary, we select the subsequence with the smallest standard deviation among the subsequences with a number of samples greater than 4000 as the CSS *I*_0_(*t*) for the next step of hypothesis testing.

#### 2.2.3. Two-Sample Kolmogorov-Smirnov Test

The two-sample KS test is used to test whether two underlying one-dimensional probability distributions differ [28]. We use the CSS *I*_0_(*t*) obtained in the previous section as a benchmark to test the rest of the *m*-1 subsequences *I_h_*(*t*) (*h* = 1, 2,…, *m* − 1) to locate all the stationary subsequences in the original GAT signal. To test whether *I_h_*(*t*) and *I*_0_(*t*) are from the same distribution, the two-sided test is chosen. In this case, the KS statistic can be calculated by [29].

(8) $$D_{KS}=\underset{x}{\sup}|F_{N_{1}}\left(x\right)-F_{N_{2}}\left(x\right)|$$

where sup is the supremum function, and $F_{N_{1}}\left(x\right)$ and $F_{N_{2}}\left(x\right)$ are the empirical distribution functions of *I_h_*(*t*) and *I*_0_(*t*), respectively. Subsequently, the significance level of an observed value of *D_KS_* is given by [30]

(9) $$P_{KS}=2\sum_{j=1}^{100}{\left(-1\right)}^{j-1}e^{-2j^{2}\lambda^{2}}$$

where $\lambda \;=\left(\sqrt{N_{KS}}+0.12+0.11/\sqrt{N_{KS}}\right)D_{KS},N_{KS}=\frac{N_{1}N_{2}}{N_{1}+N_{2}}$; *N*_1_ and *N*_2_ are the number of samples of *I_h_*(*t*) and *I*_0_(*t*), respectively. The *P_KS_* was compared with the significance level of *α* = 0.01 in this paper. Subsequently, assume null hypothesis H_0_ and alternative hypothesis H_1_.

**H_0_:**  *I_h_(t) and I_0_(t) are from the same distribution;*

**H_1_:**  *I_h_(t) and I_0_(t) are from different distributions.*

If *P_KS_* > *α*, the conclusion is that we accept the null hypothesis H_0_ under the 99% confidence level, i.e., *I_h_*(*t*) and *I*_0_(*t*) are from the same distribution, and we consider that *I_h_*(*t*) is a stationary subsequence. If *P_KS_*≤*α*, the conclusion is to reject the null hypothesis H_0_ under the 99% confidence level, i.e., *I_h_*(*t*) and *I*_0_(*t*) are from different distributions and considered that *I_h_*(*t*) is a jumping subsequence.

#### 2.2.4. Specific Steps of the HSA-KS Method

The HSA-KS method mainly includes the following steps:

Step 1: The original jumping GAT signal affected by the instantaneous disturbance torque *I_R_*(*t*) is divided into *m* non-overlapping subsequences $I_{R_{1}}\left(t\right),I_{R_{2}}\left(t\right),\cdots ,I_{R_{m}}\left(t\right)$ by HSA.

Step 2: The subsequence with the smallest standard deviation among the subsequences with sampling numbers greater than 4000 is selected as the CSS *I*_0_(*t*).

Step 3: All the stationary subsequences are determined using the two-sample KS test between CSS *I*_0_(*t*) and the remaining *m*-1 subsequences *I_h_*(*t*) (*h* = 1, 2,…, *m* − 1).

Step 4: The mean value of the rotor current is computed using the stationary subsequences, and then, the processed gyro north azimuth is calculated.

**Figure 1:**
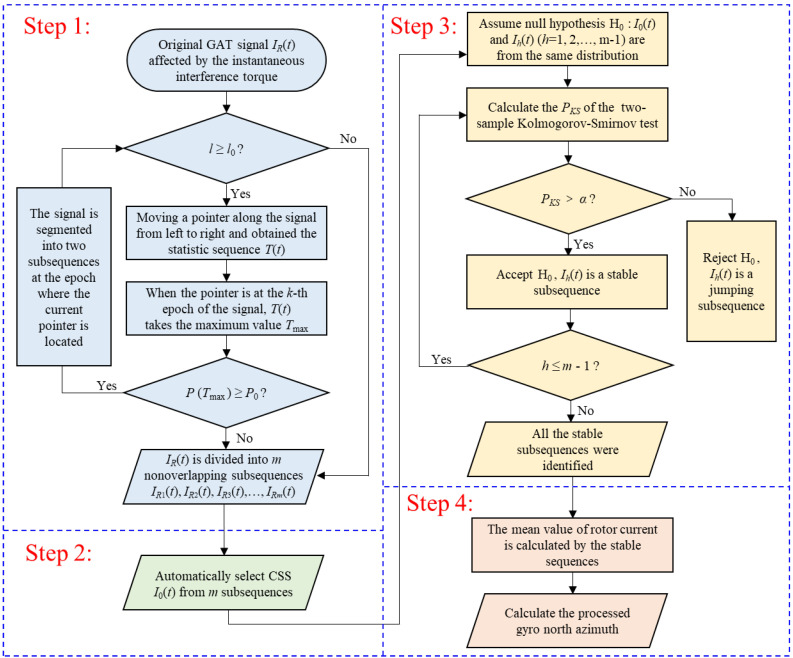
Flow chart of the HSA-KS method.

The flow chart of the specific steps of the HSA-KS method is shown in Figure 1.

### 2.3. Experimental Design

#### 2.3.1. Field Experiment

A field experiment was designed to verify the effectiveness of our method for processing the GAT signals affected by the instantaneous disturbance torque. The experimental site was located at the entrance of the 5th sub-tunnel of the Qinling water conveyance tunnel of the Hanjiang-to-Weihe River Diversion Project in Shaanxi Province, China. The tunnel has a total length of 98.30 km with a maximum burial depth of about 2000 m. The latitude of the experimental area is 39.9°.

**Figure 2:**
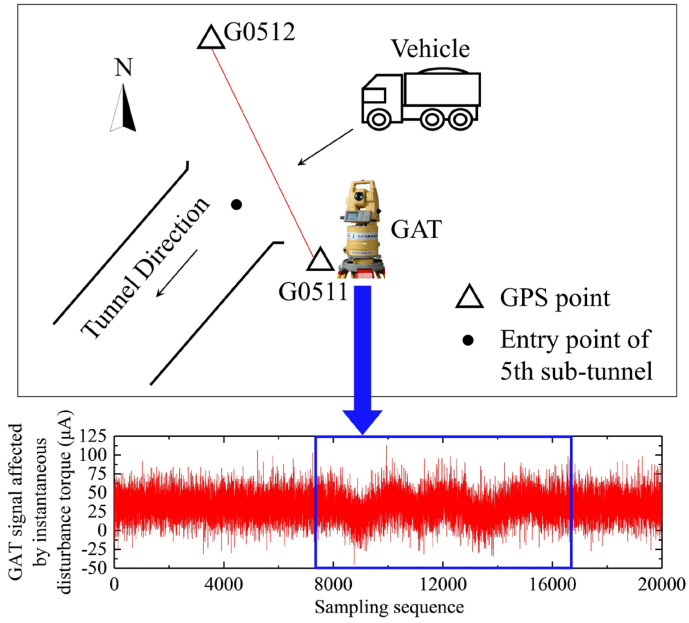
Schematic diagram of the field experiment.

Figure 2 shows a schematic diagram of north seeking using the GAT in the experimental field. The GAT was placed on the G0511 point to measure the north azimuth of the GPS baseline G0511-G0512. The accuracy of the north azimuth obtained using high-precision GPS was better than 0.5″, which was significantly higher than that of the GAT (3.5″). Therefore, the north azimuth obtained using high-precision GPS could be regarded as a relative true north azimuth to check the gyro north azimuth in our experiment. When using the GAT for north seeking, vibrations due to the passage of vehicles often occur on the nearby ground. At this time, GAT was affected by the instantaneous disturbance torque, and its measured signal exhibited a jump, as shown in Figure 2. In total, 10 sets of GAT signals affected by the instantaneous disturbance torque caused by the passing vehicles were measured on the high-precision GPS baseline.

#### 2.3.2. Three Schemes for Processing GAT Signals

In this paper, three different schemes were used to process the GAT signal affected by the instantaneous disturbance torque, and the results were compared and analyzed. The details of the three schemes were as follows:

Scheme 1: the HSA-KS method.

Scheme 2: the optimized HHT method. An optimized filtering strategy for processing the GAT signals based on the HHT proposed by Zhang et al. [14] was chosen. HHT is an effective tool for processing non-stationary signals [31]. HHT is composed of two parts: the empirical mode decomposition (EMD) and the Hilbert transform. The GAT signals can be decomposed into several intrinsic mode function (IMF) components according to the frequency because the basis functions of EMD could be derived from the signal itself. Then, applying the Hilbert transform on these IMF components, the physical instantaneous amplitude and instantaneous frequency can be obtained [32]. Based on the weighted power distribution introduced by Zhang [14], the optimal decomposed and reconstructed levels of the GAT signal were adaptively selected using the optimized HHT method.

Scheme 3: the optimized WT method. An optimized WT method for processing the GAT signals proposed by Ma et al. [10] was chosen. WT is also a method for analyzing and processing non-linear and non-stationary signals [33]. The GAT signal is divided into low-frequency and high-frequency coefficients, and the low-frequency coefficients are decomposed layer by layer, so the final signal is decomposed into a residual low-frequency coefficient and a series of high-frequency coefficients. The high-frequency coefficients are threshold de-noised by the corresponding criteria, and the wavelet coefficients after thresholding are reconstructed [10,34]. In the optimized WT method, a soft threshold function [35] was introduced by Ma et al. as the criteria to smooth the jump trend in the GAT signals and to adaptively determine the optimal wavelet decomposed level.

To analyze the stationarity of the GAT signals and to compare the processing results of the above three schemes, we introduced the autocorrelation function (ACF) and the autocorrelogram, which is a widely used method for testing the stationarity of the signals. The ACF reveals how the correlation between any two samples of the signal varies with their interval [36]. The ACF of the GAT signal $I_{R}\left(t\right)\;={\left(I_{R_{1}},I_{R_{2}},\cdots ,I_{R_{N}}\right)}^{T}$ is defined as

(10) $$\rho_{k}=\frac{\sum_{t=1}^{N-k}(I_{R}\left(t\right)-\overset{¯}{I_{R}})(I_{R}\left(t+k\right)-\overset{¯}{I_{R}})}{\sum_{t=1}^{N}{\left(I_{R}\left(t\right)-\overset{¯}{I_{R}}\right)}^{2}}$$

where *N* is the total number of samples, i.e., *N* = 20,000. $\overset{¯}{I_{R}}$ is the mean value of $I_{R}\left(t\right)$, and *k* is called the lag order. From Equation (10), we can conclude that $\rho_{0}=1$ and $-1\leq \rho_{k}\leq 1$. By calculating the ACF value corresponding to the lag order *k* from 0 to *n* using the above equation and plotting it, the autocorrelogram can be obtained. As the lag order *k* in Equation (10) increases, the ACF in the autocorrelogram of the stationary signal drops rapidly to zero and fluctuates randomly around the horizontal axis. However, the ACF in the autocorrelogram of the non-stationary signal drops more slowly and does not approach zero. This is the criterion of the stationarity test using the autocorrelation function and the autocorrelogram [37,38].

The final goal of gyro signal processing is to improve the north-seeking accuracy of the GAT. To compare the north-seeking accuracy after processing using the three schemes mentioned above, the absolute difference between the gyro and high-precision GPS north azimuths *D* was introduced into this study. The *D*-value can be given by

(11) $$D=\left|\alpha_{T}-\alpha_{0}\right|$$

where $\alpha_{T}$ is the gyro north azimuth and $\alpha_{0}$ is the high-precision GPS north azimuth.

## 3. Results and Discussion

### 3.1. Typical GAT Signals

**Figure 3:**
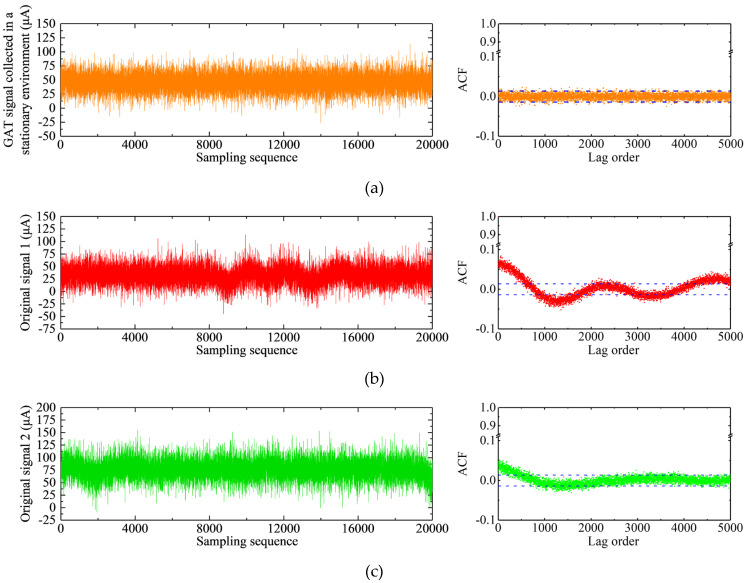
Time-series diagrams and the autocorrelograms of the original GAT signals: (**a**) GAT signal collected in a stationary environment; (**b**) typical jumping signal 1; (**c**) typical jumping signal 2.

We selected two typical jumping signals measured in the experimental area as examples. The time-series diagrams and the autocorrelograms of the original GAT signals were illustrated in Figure 3.

In Figure 3, the time-series diagram of the signal is shown on the left and its corresponding autocorrelogram is shown on the right. In the time series of the signal, the abscissa and the ordinate represented the sampling epoch and the GAT rotor current value, respectively. In the autocorrelogram, the abscissa and the ordinate represented the lag order and the ACF value, respectively. The area between the two blue dashed lines was the 95% confidence interval, indicating whether the autocorrelations were significantly different from zero. The ACF was considered to be zero when it was within this interval [39]. In Figure 3a, the time series diagram exhibited the trend of the GAT signal measured in a stationary environment that remained stable and accompanied by high-frequency random noise. This type of signal was referred to as “the steady GAT signal” by some scholars [10,14,15,40]. The final gyro north azimuth was calculated by averaging *N* = 20,000 sets of rotor current values (Equations (2) and (3)). Since the number of samples was large enough, the effect of high-frequency random noise in the signal on the accuracy of north seeking can be neglected [9]. The autocorrelogram of the signal in Figure 3a suggested that the steady GAT signal can be considered as a stationary signal [37].

As demonstrated in Figure 3b, the time-series diagram of signal 1 remained stationary at the beginning of sampling and was affected by the passing vehicle at about 8000 epochs. At this point, the bias correction mechanism of the GAT was activated. In this correction process, the GAT signal revealed a quasi-periodic fluctuation. Finally, the signal gradually returned to its original stable state at about 16,000 epochs. As can be seen from the autocorrelogram in Figure 3b, the ACF did not fluctuate within the two blue dashed lines, indicating that signal 1 was non-stationary [38].

Figure 3c indicated that the time-series diagram of signal 2 was disturbed by the passing vehicle at the beginning of sampling and recovered to a stable state at about 6000 epochs by the bias correction mechanism of the GAT. When the sampling was continued to about 19,000 epochs, the signal was disturbed by the second passing vehicle and exhibited a deviation. However, the bias correction process needed to last for a period of time. Before the end of the signal sampling, the correction process has not been completed and the signal has not yet returned to its stable state. The autocorrelogram in Figure 3c revealed that this signal was also non-stationary.

Figure 3b,c illustrated that signals 1 and 2 were offset in some sampling subsequences and exhibited non-stationary characteristics due to the influence of the instantaneous disturbance torque. These jumping subsequences biased the results of the mean algorithm (Equations (2) and (3)), which reduced the north-seeking accuracy of the GAT. Therefore, it is necessary to process this systematic error. Numerous studies have verified that the steady GAT signal (Figure 3a) was the ideal type [9,10,15]. Therefore, after processing the jumping GAT signals, we expect to acquire a signal and the corresponding autocorrelogram similar to this type.

### 3.2. Signal Processing Results

After processing the two typical jumping GAT signals, the results were observed to be significantly different. Therefore, the processing results of the two typical jumping signals were discussed in detail individually below.

#### 3.2.1. Signal 1 Processing Result

**Figure 4:**
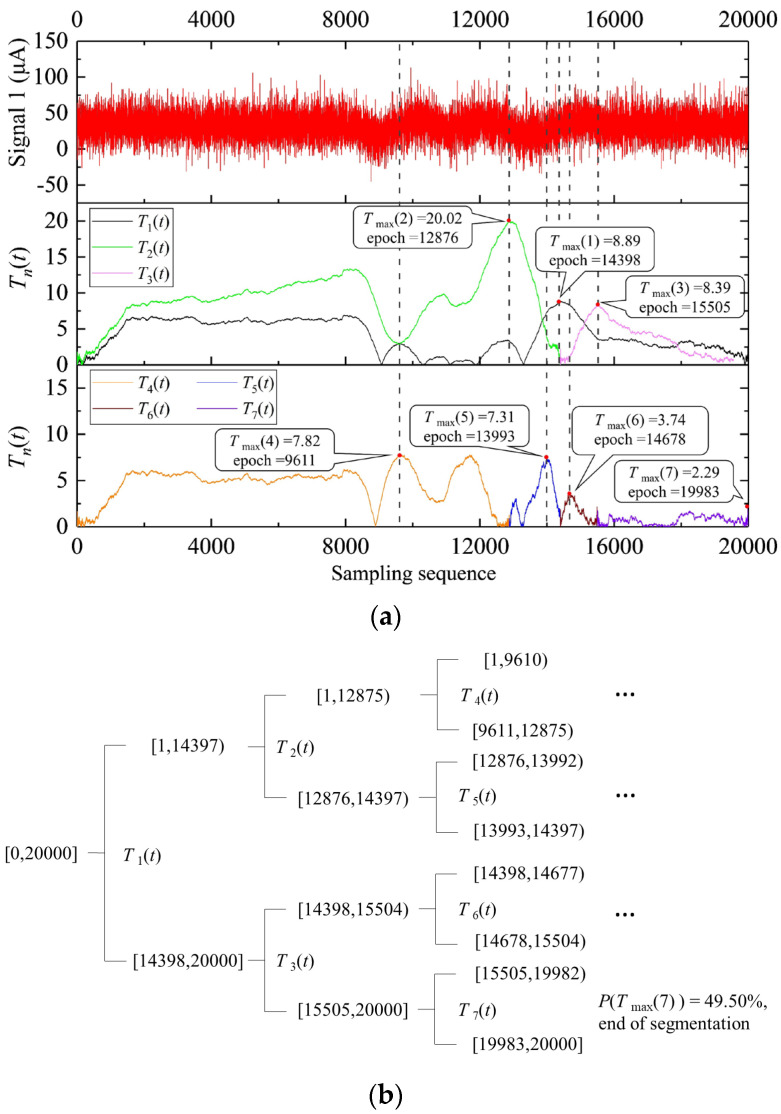
First seven segmentation processes of signal 1 using the HSA-KS method: (**a**) time series of signal 1 and the first seven *T_i_*(*t*); (**b**) dendrogram of the segmentation processes.

The first seven segmentation processes of signal 1 using the HSA-KS method are shown in Figure 4. The statistics sequence *T*(*t*) of the *i*-th segmentation was denoted as *T_i_*(*t*). The first seven *T_i_*(*t*) were exhibited in Figure 4a with different color curves. The position of each *T_max_*(*i*) was marked with red dots, and the corresponding change points were marked with gray dotted lines. Meanwhile, the value of each *T_max_*(*i*) and the epochs of each change point were also marked in Figure 4a. Figure 4b showed the dendrogram of the first seven segmentation processes. The curves of each statistic sequence *T_i_*(*t*) (i = 1, 2, ..., 7) in Figure 4a all corresponded to one another in Figure 4b. In the first segmentation, the statistic sequence *T*_1_(*t*) of the original signal achieves the maximum value *T_max_*(1) = 8.89 at the 14,398th epoch (Figure 4a). Since *P*(T*_max_*(1))>*P*_0_ (Equation (6)), the original signal was divided into two subsequences: [1, 14,397) and [14,398, 20,000] (Figure 4b). In the second segmentation, the statistic sequence *T*_2_(*t*) of the subsequence [1, 14397) achieves a maximum value *T_max_*(2) = 20.02 at the 12,876th epoch (Figure 4a). Since *P*(*T_max_*(2))>*P*_0_ (Equation (6)), the subsequence [1, 14,397) was divided into two subsequences: [1, 12,875) and [12,876, 14,397) (Figure 4b). All the segmentations after the third time were performed in the same way.

**Figure 5:**
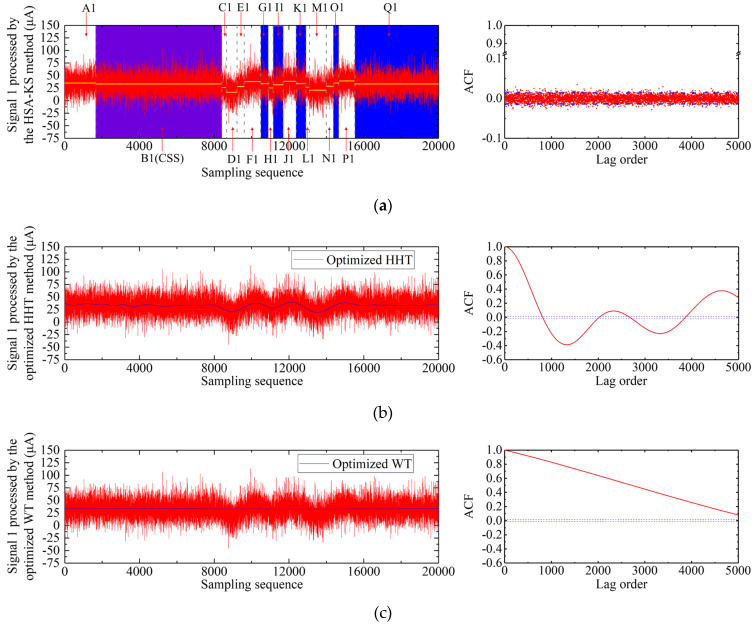
Results of signal 1 processed using the three different schemes: (**a**) HSA-KS method; (**b**) optimized HHT method; (**c**) optimized WT method.

Figure 4 demonstrated that after the first seven segments, the original signal 1 was divided into seven subsequences. In the seventh segmentation, the subsequence was not segmented since *P*(*T_max_*(7)) < *P*_0_ (Figure 4b). Other subsequences continued to be segmented until all *P*(*T_max_*(*i*)) < *P*_0_. The final HSA-KS processing result of signal 1 was shown in Figure 5a.

**Figure 6:**
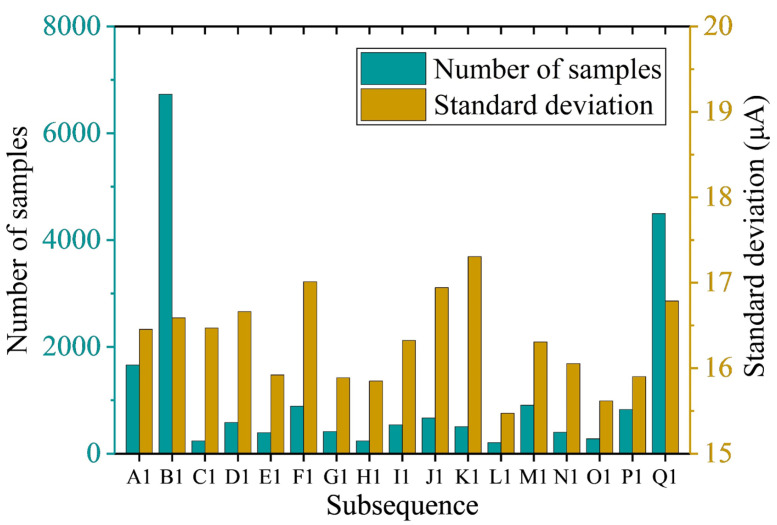
Number of samples and standard deviation of each subsequence segmented by the signal 1.

As shown in Figure 5a, after 16 segmentations, the original signal 1 was divided into 17 subsequences with different mean values. The 17 subsequences were defined as A1, B1, C1, …, and Q1 from left to right and are marked in Figure 5a. The mean values of each subsequence are displayed with yellow horizontal lines (Figure 5a). The number of samples and standard deviation of each subsequence are illustrated in Figure 6.

**Figure 7:**
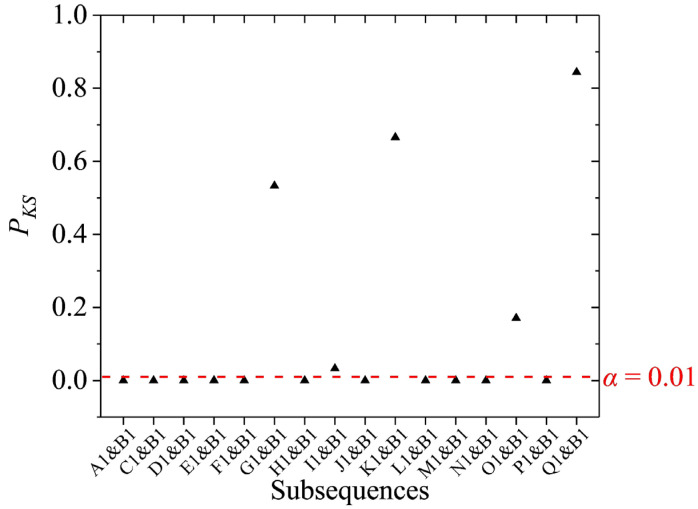
Results of the two-sample KS test of the subsequences segmented by the signal 1.

According to the judgment criteria of CSS in Section 2.3.2 and the results in Figure 6, both the number of samples of subsequences B1 and Q1 was greater than 4000. Since the standard deviation of B1 was less than Q1 (Figure 6), B1 was selected as the CSS and marked with a purple background in Figure 5a. The remaining subsequences were performed on the two-sample KS test with B1 in turn, and the results are presented in Figure 7.

Figure 7 showed that the subsequences G1, I1, K1, O1, and Q1 passed the two-sample KS test (*P_KS_* was greater than 0.01) and were marked with a blue background in Figure 5a. Therefore, among the subsequences segmented by the signal 1, B1, G1, I1, K1, O1, and H1 were the stationary subsequences. Figure 5a displayed that the autocorrelogram of the signal 1 processed by the HSA-KS method was similar to that of the steady GAT signal (Figure 3a), indicating that the signal can be considered as a stationary signal. This result suggested that the jumps caused by the instantaneous disturbance torques in the signal 1 can be accurately identified and eliminated using the HSA-KS method. The number of samples of the remaining stationary subsequences in the processed signal 1 was 12,973. The processed gyro north azimuth was calculated by the mean value of these identified stationary subsequences, and the results of the *D*-values before and after processing were demonstrated in Table 1. Although the number of samples of signal 1 decreased after processing, all of the segments removed by the HSA-KS method were contaminated signal subsequences. Therefore, Table 1 illustrated that after the processing using the HSA-KS method, the *D*-value of the signal 1 decreased from 9.2″ to 3.5″, indicating that the processed gyro north azimuth was closer to the high-precision GPS north azimuth. This result could be explained by the fact that the jumping subsequences caused by the passing vehicle in the original GAT signal were eliminated after processing. Therefore, the processed gyro north azimuth was no longer affected by the error caused by the instantaneous disturbance torque, and the external coincidence accuracy of north-seeking was improved. 

The processing result of signal 1 using the optimized HHT method was demonstrated in Figure 5b. Signal 1 was decomposed into 11 IMF components and one residual component by empirical mode decomposition. The final filtered signal was reconstructed by the 8th to 11th IMF components and the residual component (Figure 5b). The time-series diagram indicated that the overall trend of the processed signal 1 did not change, and its high-frequency random noise was eliminated. The autocorrelogram of the signal 1 after processing had a similar trend to that before processing (Figure 3b), indicating that it was still non-stationary. Table 1 demonstrated that the *D*-value only changed by 0.9″ after processing. The explanation for this result could be that the final gyro north azimuth was determined by the mean value of *N* = 20,000 sets of rotor current values (Equations (2) and (3)), which makes the high-frequency random noise have a minor influence on the final gyro azimuth [9]. Our results suggested that the optimized HHT method cannot eliminate the error caused by the instantaneous disturbance torque in signal 1; the absolute difference between the processed gyro north azimuth and the true value was still 10.1″ (Table 1).

Figure 5c illustrated the processing result of signal 1 using the optimized WT method. The Symlets wavelet function with a vanishing moments number of 10 was selected as the wavelet basis function in the method [10]. The wavelet decomposed level of signal 1 was adaptively selected as level 14 using the method. The time-series diagram in Figure 5c exhibited that the waveform of signal 1 entirely changed after processing due to choosing the high wavelet decomposed level. The low-frequency content in signal 1 was excessively smoothed, resulting in signal distortion. However, Figure 5c also revealed that the jumping trend in signal 1 was eliminated after filtering. This result was similar to the results in reference [10]. The *D*-value in Table 1 reduced from 9.2″ to 3.3″ after processing, indicating that the external coincidence accuracy of gyro north seeking has improved. The autocorrelogram showed that the ACF was positive and slowly decreased as the lags increased. This result suggested that the signal 2 processing by the optimized WT method was a non-stationary signal with a trend [38]. Since the original trend of signal 1 was changed after processing by the optimized WT method, the effectiveness and stability of this method need to be further verified.

#### 3.2.2. Signal 2 Processing Result

**Figure 8:**
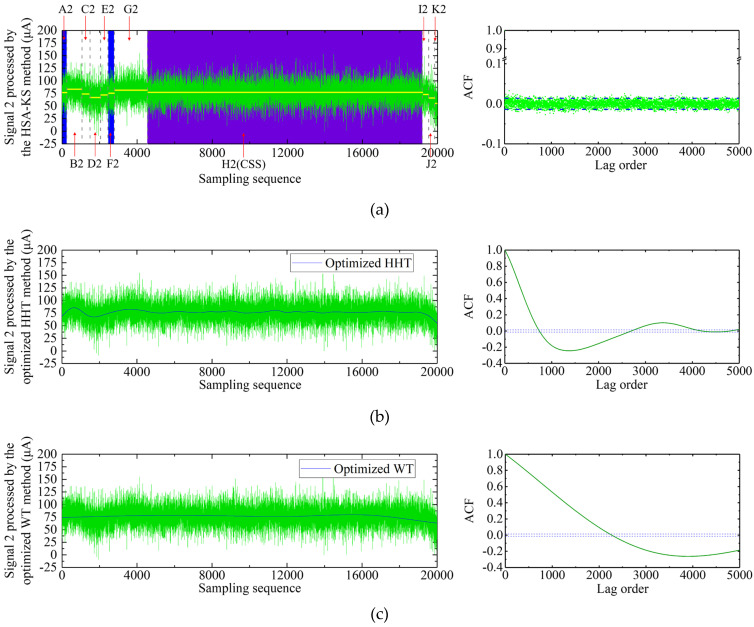
Results of signal 2 processed using the three different schemes: (**a**) HSA-KS method; (**b**) optimized HHT method; (**c**) optimized WT method.

The processing results of signal 2 through different schemes were demonstrated in Figure 8. Figure 8a showed the processing result of signal 2 using the HSA-KS method. After 10 segmentations, the original signal 2 was divided into 11 subsequences. The 11 subsequences were defined as A2, B2, C2, …, and K2 from left to right and are marked in Figure 8a. Among the subsequences, H2 was the CSS (Figure 8a, purple background). The subsequences A2 and F2 were the stationary subsequences determined by the two-sample KS test (Figure 8a, blue background). Figure 8a displayed that the autocorrelogram of signal 1 processed by the HSA-KS method was similar to that of the stationary GAT signal (Figure 3a) and signal 1 (Figure 5a), indicating that the signal can be considered as a stationary signal. This result further verified that the jumps caused by the instantaneous disturbance torques in the GAT signal can be clearly removed using the HSA-KS method. The number of samples of the remaining stationary subsequences in the processed signal 2 was 15,292. Table 1 illustrated that the *D*-value decreased from 3.5″ to 1.4″ after processing, which indicated that the processed gyro north azimuth was closer to the true value. This result showed that although the total number of samples of the processed signal 2 has decreased, the north-seeking accuracy of the GAT was improved because all the eliminated sequences were contaminated jumping subsequences. The autocorrelograms of signals 1 and 2 suggested that the jumping subsequences caused by the instantaneous disturbance torque in the GAT signals can be detected and eliminated accurately and automatically using the HSA-KS method. Our results demonstrated that the *D*-values of signals 1 and 2 decreased to 3.5″ and 1.4″, respectively, after processing by the HSA-KS method (Table 1), indicating that the north-seeking accuracy and the anti-interference ability of the GAT have been improved. This result also showed that although the total number of samples of signals 1 and 2 decreased after processing by the HSA-KS method, the number of retained samples could still guarantee the nominal north-seeking accuracy of the GAT.

Figure 8b illustrated the processing result of signal 2 using the optimized HHT method. Signal 2 was decomposed into 11 IMF components and one residual component. The final filtered signal was reconstructed by the 8th to 11th IMF components and one residual component. Figure 8b exhibited that the overall trend of the processed signal 2 did not change, while the high-frequency random noise was eliminated, which was similar to the result of the signal 1 (Figure 5b). The autocorrelogram showed that the processed signal was still non-stationary due to the fact that the jumps in the signal have not been removed. Table 1 indicated that the *D*-value raised from 3.5″ to 4.0″ after processing, with a change of only 0.5″. This result could also be explained by the fact that the high-frequency random noise in the GAT signal only had a minor effect on the final gyro azimuth. The processing results of signals 1 and 2 demonstrated that the high-frequency random noises in the GAT signals can be effectively removed by the optimized HHT method, but the jumps caused by the instantaneous disturbance torque cannot be eliminated.

The processing result of signal 2 using the optimized WT method is exhibited in Figure 8c. The wavelet decomposed level of signal 2 was adaptively selected as level 12. The time-series diagram in Figure 8c showed that the jumping trend at the sampling start part (epoch 1 to 4548) in signal 2 has been rectified after processing. Before the end of the signal 2 sampling, the bias correction process of the GAT has not been completed. The signal has not returned to its original stable state and shows a downward jumping trend at the end of the sampling (epoch 19,225 to 20,000). However, Figure 8c reveals that this downward jumping trend in signal 2 was not eliminated but even amplified after processing using the optimized WT method. This result was reflected in the increase in the *D*-value from 3.5″ to 7.5″ after processing (Table 1), which indicated that the external coincidence accuracy of north seeking was decreased. As the jumps in the processed signal were not completely removed, the autocorrelogram shows that the processed signal was still non-stationary (Figure 8c). Our results suggested that the GAT signals that were affected by the instantaneous disturbance torque and then restored to their original stable state by the bias correction mechanism can be rectified the jumping trends and improve the north-seeking accuracy using the optimized WT method (e.g., signal 1, Figure 5c). However, using the optimized WT method to process the GAT signals that were affected by the instantaneous disturbance torque and did not return to the original stable state by the bias correction mechanism may amplify the jumping trends and reduce the north-seeking accuracy (e.g., signal 2, Figure 8c). Therefore, the reliability of the optimized WT method for processing the jumping GAT signal was less than that of the HSA-KS method.

### 3.3. Results of the D-Values

**Figure 9:**
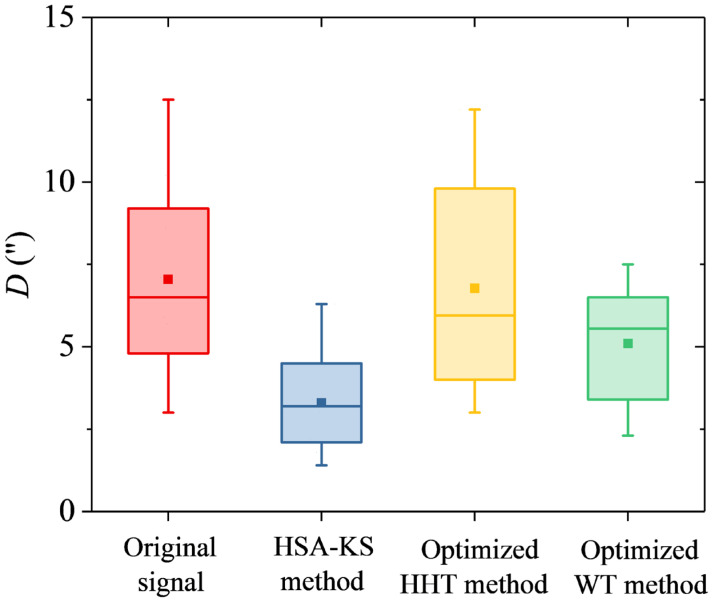
Boxplot of the *D*-values processed by the three schemes.

In our experiment, a total of 10 sets of data affected by the instantaneous disturbance torques were collected in the experimental group. Each of the GAT signals in the experimental group contained *N* = 20,000 sets of current data. Meanwhile, the gyro north azimuths calculated from the data in the experimental group all had corresponding relative true values (the high-precision GPS north azimuths) for comparison. In the three schemes for processing the GAT signals in the experimental group, the gyro north azimuths processed by the HSA-KS method were calculated using the mean values of the detected stationary subsequences in the GAT signals (Equations (2) and (3)). For the optimized HHT and the optimized WT method, the processed gyro north azimuths were calculated by the mean values of the filtered GAT signals using Equations (2) and (3). After calculating the processed gyro north azimuths using each of the GAT signals in the experimental group, the final *D*-values were subsequently computed by Equation (11). The boxplot of the *D*-values processed by the above three schemes is presented in Figure 9, and the statistical results are shown in Table 2. In Figure 9, the boxplot consisted of a box and a set of whiskers (lines extending from the boxes). The highest and lowest horizontal lines of the boxplot represented the maximum and minimum of the dataset, respectively. The box was drawn from the 25th to 75th percentile, with a square-dot representing the mean value and a horizontal line representing the median in the middle.

Experimental results indicated that the *D*-values of the GAT signals affected by the instantaneous disturbance torque achieved an average gain effect of 53.5% after processing using the HSA-KS method, which was the highest among the three schemes. The *D*-value significantly decreased from 7.1″ to 3.3″ on average (*p* < 0.05, *n* = 10, where *p* represents the *p*-value and *n* represents the number of the *D*-values). The explanation for this result could be that the jumping subsequences in the GAT signals caused by the instantaneous disturbance torque were automatically and accurately detected and eliminated after processing using the HSA-KS method. Therefore, the external coincidence accuracy and the environmental adaptability of the GAT were improved.

**Figure 10:**
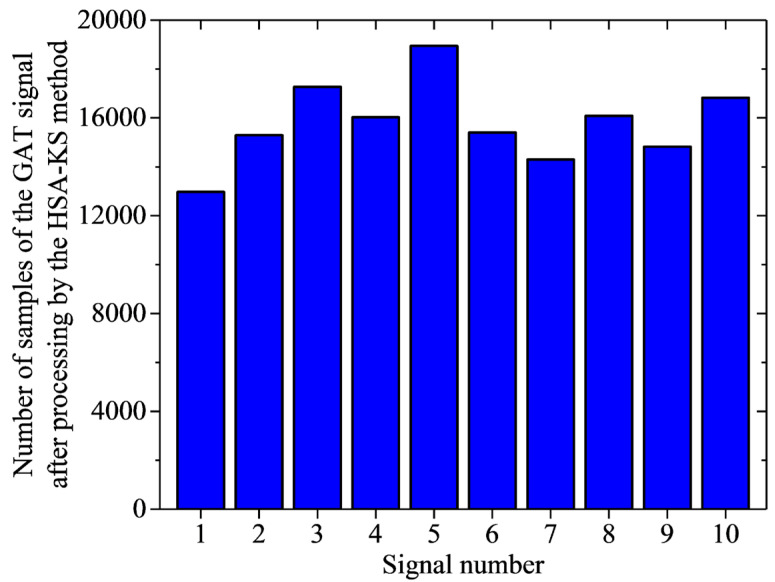
Number of samples of each signal in the experimental group after processing by the HSA-KS method.

Figure 10 illustrates the number of samples of the 10 sets of the jumping GAT signals collected in our experiment after processing by the HSA-KS method. In the bar chart of Figure 10, the horizontal and vertical axis indicated the signal number and the number of samples remaining in the processed signal, respectively. Among them, the signal with the largest number of samples after processing was signal 5, which had 18,950 samples. The signal with the least number of samples after processing was signal 1, which had 12,973 samples. Although signal 1 had the largest reduction in the number of samples after processing, its *D*-value decreased from 9.2″ to 3.5″ after processing (Table 1). This result demonstrated that the north-seeking accuracy of the GAT was improved after processing because of the automatic and accurate detection and elimination of the contaminated jumping subsequences in signal 1 by the HSA-KS method. In conclusion, the number of samples in the processed signal is sufficient to guarantee the nominal north-seeking accuracy of the GAT as long as it is larger than the number of samples of signal 1 (12,973 samples). This conclusion was also verified in Figure 9 and Table 2, where the *D*-value was improved from 7.1″ to 3.3″ with a gain effect of 53.5% after processing by the HSA-KS method.

Our results revealed that there was no significant difference between the *D*-values before and after the GAT signals processed by the optimized HHT method in our experiment (*p* < 0.1, *n* = 10). This result could be explained by the fact that after processing the GAT signal using the optimized HHT method, only the high-frequency random noise was removed, while the jumps caused by the instantaneous disturbance torque cannot be eliminated (Figure 5b and Figure 8b). However, the final gyro azimuth was calculated by averaging *N* = 20,000 sets of rotor current values (Equations (2) and (3)), which makes the high-frequency random noise have a minor impact on the final north-seeking results [9]. 

Our results suggested that the *D*-value of the GAT signals achieved average gain effect of 28.2% after processing by the optimized WT method in our experiment. The results indicated that the *D*-value significantly decreased from 7.1″ to 5.1″ on average (*p* < 0.1, *n* = 10). The rationale for this improvement was that in the GAT signals that were affected by the instantaneous disturbance torque and then restored to their original stable state by the bias correction mechanism, the errors caused by the instantaneous disturbance torque can be filtered after processing by the optimized WT method (e.g., signal 1, Figure 5c). However, using the optimized WT method to process the GAT signals that were affected by the instantaneous disturbance torque and did not return to the original stable state by the bias correction mechanism may lead to the jumping trends being exacerbated (e.g., signal 2, Figure 8c). Among the 10 sets of GAT signals collected in our experiment, the percentage of signals similar to signal 1 was 70%, and the percentage of signals similar to signal 2 was 30%. The overall improvement in the *D*-value after processing by the optimized WT method may be related to the fact that the larger proportion of the signals was similar to signal 1 in our experiment. Therefore, the reliability of the optimized WT method for processing the jumping GAT signal was less than that of the HSA-KS method.

## 4. Conclusions

From the above results and analysis, we can conclude that the HSA-KS method was the most efficient method for processing the GAT signals affected by the instantaneous disturbance torque in the three schemes. In our experiment, the *D*-value of the GAT signal affected by the instantaneous disturbance torque improved by 53.5% on average using the HSA-KS method, which was the highest among the three schemes. Our results from the autocorrelograms indicated that the jumps in the GAT signals can be automatically and accurately eliminated using the HSA-KS method. The HSA-KS method was suggested for processing the jumping GAT signals. The north-seeking accuracy and the environmental adaptability of the GAT affected by the instantaneous disturbance torque can be improved using the HSA-KS method.

After addressing the issue of the degradation of north-seeking accuracy of the GAT by the instantaneous disturbance torque, further research needs to be conducted on how to solve the problem of the GAT affected by continuous disturbance torque (primarily wind vibration). Therefore, the next step is to perform quantitative experiments in the continuous wind vibration environments and to establish mathematical models between the vibrations and the deviated GAT signals. Corresponding compensation algorithms should be provided to further improve the north-seeking accuracy of the GAT.

## Figures and Tables

**Table 1:** *D*-values (″) of typical jump signals before and after processing by the three schemes.

Scheme	Original Signal	HSA-KS Method	Optimized HHT Method	Optimized WT Method
Signal 1	9.2	3.5	10.1	3.3
Signal 2	3.5	1.4	4.0	7.5

**Table 2:** Mean value of the *D-*values (″) and the corresponding improvement of the GAT signals processed by the three schemes.

Scheme	Original Signal	HSA-KS Method	Optimized HHT Method	Optimized WT Method
Mean value	7.1	3.3	6.8	5.1
Improvement	—	53.5%	—	28.2%

## Data Availability

The data that support the findings of this study are available upon reasonable request from the authors.

## Abbreviations

ACF	autocorrelation function
CSS	categorical stationary subsequence
EMD	empirical mode decomposition
GAT	maglev gyro total station
GPS	global positioning system
HHT	Hilbert-Huang transform
HSA	heuristic segmentation algorithm
IMF	intrinsic mode function
KS	Kolmogorov-Smirnov
RMSE	root mean square error
WT	wavelet transform
$\alpha_{T}$	gyro north azimuth
*N*	total number of the current samples
$I_{R_{i}}$	collected real-time rotor current value of the torquer
$I_{S_{i}}$	collected real-time stator current value of the torquer
*T_i_*(*t*)	statistics sequence in the HSA-KS method of the *i*-th segmentation
*T_max_*(*i*)	maximum value of *T_i_*(*t*)
*P*(*T*_max_)	the statistical significance
*l*	the total number of samples of the signal to be segmented
*I_0_(t)*	signal sequence of the CSS
*I_h_(t)*	subsequence with CSS for the two-sample KS test
$D_{KS}$	KS statistic
*P_KS_*	significance level of $D_{KS}$
*α*	significance level
*m*	number of the subsequences segmented from the original signal
A1, B1, C1, …, and Q1	subsequences segmented by the signal 1
A2, B2, C2, …, and K2	subsequences segmented by the signal 2
*D*-value	absolute difference between the gyro and high-precision GPS north azimuths
